# The Role of Ganyu in Formation of Liver Depression and Spleen Deficiency Syndrome: Analysis From Gut Microbiota

**DOI:** 10.1155/grp/2125189

**Published:** 2025-09-09

**Authors:** Yinghan Wang, Tingting Pan, Nan Hu, Guangtai Gao, Xiaorui Jia, Yinghui Zhang, Chunru Song, Chunying Yin, Yuling Liu

**Affiliations:** ^1^Key Laboratory for Research and Development of Chinese Traditional Medicine of Hebei Province, Institute of Chinese Materia Medica, Chengde Medical College, Key Research Laboratory of Anti-Dementia of Traditional Chinese Medicine of Hebei Province, Chengde, Hebei, China; ^2^Institute of Chinese Materia Medica, Chengde Medical College, Chengde, Hebei, China; ^3^Department of Traditional Chinese Medicine, Chengde Medical College, Chengde, Hebei, China

**Keywords:** 16S rRNA sequencing, diarrhea-dominant irritable bowel syndrome, gut microbiota, liver depression and spleen deficiency syndrome, visceral sensitivity

## Abstract

**Background:** Diarrhea-predominant irritable bowel syndrome (D-IBS) is a clinically common functional intestinal disease, classified into “diarrhea,” “abdominal pain,” and “depression syndrome” categories according to traditional Chinese medicine (TCM). The exact pathogenesis of D-IBS is still not fully understood. Gut microbiota regulates gastrointestinal nerve, endocrine, and immune functions and maintains gastrointestinal homeostasis through interaction with the brain–gut axis. In this study, we assessed the changes in gut microbiota in a D-IBS rat model with liver depression, spleen deficiency, and liver depression and spleen deficiency syndrome. We also discussed the biological basis of liver depression and spleen deficiency syndrome and the associations among the three syndromes from the perspective of gut microbiota.

**Methods:** Ninety rats were divided into nine groups randomly: normal group (ZC), spleen deficiency syndrome groups (four PX groups), liver depression syndrome groups (two GY groups), and liver depression and spleen deficiency syndrome groups (two GYPX groups). The abdominal wall withdrawal reflex (AWR) test detected visceral sensitivity, while changes in gut microbiota were analyzed using 16S rRNA sequencing.

**Results:** The visceral sensitivity of rats in the model group was significantly higher than that in the ZC group, and the visceral sensitivity of the GYPX groups was significantly higher compared to the PX and GY groups. 16S rRNA sequencing analysis showed that the D-IBS model gut microbiota's species number, alpha diversity, and beta diversity were changed; the Bacteroidota increased, and the Firmicutes decreased in the model group. The abundance of pathogenic bacteria, such as Bacteroidales, significantly increased in the GYPX groups compared to other groups.

**Conclusion:** Oral administration of senna combined with restraint stress had different effects on visceral hypersensitivity, gut microbiota composition, and metabolic pathways in rats with D-IBS liver depression and spleen deficiency syndrome, and the liver depression factors play an important role in the pathogenesis of liver depression and spleen deficiency syndrome in D-IBS.

## 1. Introduction

Diarrhea-predominant irritable bowel syndrome (D-IBS) is a clinically common functional intestinal disease, classified into “diarrhea,” “abdominal pain,” and “depression syndrome” categories according to traditional Chinese medicine (TCM). The exact pathogenesis of D-IBS is still not fully understood. Recent studies suggested a close association between D-IBS and visceral hypersensitivity, increased intestinal permeability, low-level inflammation of the intestine, intestinal motor function, and gut microbiota imbalance [1]. Gut microbiota regulates gastrointestinal nerve, endocrine, and immune functions and maintains gastrointestinal homeostasis through interaction with the brain–gut axis. Studies have found that the imbalance in gut microbiota affects intestinal immunity and intestinal smooth muscle function, leading to gastrointestinal dysfunction, which eventually results in D-IBS symptoms [2–4]. Also, preliminary experimental results of the research group showed that the formation process of the liver depression spleen deficiency syndrome, which is considered the most common pathogenesis of D-IBS in TCM [5], is closely related to changes in intestinal motility, brain–gut peptides such as 5-hydroxytryptamine (5-HT), corticotropin-releasing hormone (CRH), and related receptors. Yet, no significant pathological changes in the intestinal mucosa have been found during this process.

Previous studies have investigated the relationship between D-IBS and gut microbiota from the perspective of Western medicine, whereas the present study innovatively combined the liver depression and spleen deficiency syndromes of TCM and D-IBS to fill the gap in the study of gut microbiota under specific syndromes, which is more in line with the characteristics of TCM's diagnosis and treatment. In addition, most of the previous studies focused on the structural composition of gut microbiota, but the present study not only analyzed the structural changes of the microbiota but also combined the core pathological mechanism of liver depression and spleen deficiency syndromes in Chinese medicine and explored the diversity and structural composition of gut microbiota and the association between the three syndromes of spleen deficiency (oral administration of senna), liver depression (binding stress), and liver depression and spleen deficiency (combination of oral administration of senna and binding stress), so as to provide a clear explanation of liver depression and spleen deficiency and D-IBS in the context of gut microbiota. This study provides new ideas for the interpretation of the nature of liver depression and spleen deficiency syndrome from the perspective of gut microbiota.

Therefore, in this experiment, we replicated the rat model of D-IBS with liver depression and spleen deficiency syndrome, set up the D-IBS with spleen deficiency syndrome and D-IBS with liver depression syndrome at the same time, and detected the changes in the gut microbiota of the D-IBS model rats by using 16S rRNA sequencing to investigate the biological basis of the liver depression and spleen deficiency syndrome of D-IBS as well as the correlation between the three from the viewpoint of gut microbiota.

## 2. Materials and Methods

### 2.1. Animals

A total of 90 male SD rats, SPF grade, with a body mass of 200 ± 20 g, were purchased from Beijing Huafukang Biotechnology Co., Ltd (the production license number of experimental animals was SCXK [Beijing] 2019-0008, and the animal quality certificate number was 110322200101506868). Ten rats were raised per cage at the Experimental Animal Center of Chengde Medical College in an environment with a temperature of 22°C ± 2°C, relative humidity of 50% ± 1%, and a light/dark cycle of 12/12 h. All animal studies (including the mouse euthanasia procedure) were done in compliance with the regulations and guidelines of Chengde Medical College institutional animal care and conducted according to the AAALAC and the IACUC guidelines (the ethical batch number was CDMULAC-20201008-003).

### 2.2. Drugs

Senna leaf (Lot Number 20200409), in accordance with the 2020 edition of the Chinese Pharmacopoeia, was provided by Beijing Bencao Fangyuan Pharmaceutical Group Co., Ltd.

### 2.3. Instruments

The instruments used are as follows: MF MS105DU NewClassic electronic balance (Mettler Toledo, Switzerland), Wonbio-96E freeze grinding machine (Wonbio, Shanghai, China), SBL-10DT ultrasonic cleaning machine (Scientz, Ningbo, China), DW-86L390 ultralow temperature refrigerator (Aucma, Qingdao, China), centrifuge 5430R Frozen centrifuge (Eppendorf, Germany), and Illumina NovaSeq 6000 platform (Illumina, United States).

### 2.4. Preparation of Senna Leaf Decoction

The senna leaves were put into a 5000-mL beaker and soaked in 10 times the amount of water for 0.5 h, heated and boiled twice, 10 min for the first time and 5 min for the second time, then combined the two filtrates, and filtered with two layers of gauze. The filtrate was freeze-dried by freeze dryer and redissolved with distilled water to a final concentration of 0.6 g crude drug/mL. Samples were then sealed and stored in the refrigerator. Before use, samples were heated in a water bath at 25°C.

### 2.5. Rat Model

The rats were divided into 9 groups according to the random number table (10 rats per group): normal group (ZC), spleen deficiency syndrome D-IBS group (PX1, PX2, PX3, and PX4), liver depression syndrome D-IBS group (GY1 and GY2), and liver depression and spleen deficiency syndrome D-IBS group (GYPX1 and GYPX2). To induce the D-IBS model with spleen deficiency syndrome, rats were given senna leaf decoction (6 g crude drug/kg) by gavage once a day for 1 week (PX1), 2 weeks (PX2), 3 weeks (PX3), and 4 weeks (PX4), respectively; to induce the D-IBS model with liver depression syndrome, rats were bound with transparent tape on the front upper limbs, chest, and shoulder for 2 h, once a day, for 1 week (GY1) and 2 weeks (GY2), respectively; to establish a D-IBS model with liver depression and spleen deficiency syndrome, rats were given senna leaf decoction (6 g crude drug/kg) by gavage once a day for 2 weeks. From the 3rd week, the front upper limbs, chest, and shoulder of the rats were bound with transparent tape lasting for 2 hours, once a day, for 1 week (GYPX1) and 2 weeks (GYPX1), respectively. The normal group was given the same volume of normal saline.

### 2.6. Abdominal Withdrawal Reflex (AWR) Test

Visceral sensitivity was tested by the AWR test. Blind observers measured the AWR response, assessed according to the following scale: 0, *no behavioral response*; 1, *brief head movement followed by immobility*; 2, *contraction of the abdominal muscles*; 3, *lifting of the abdomen*; and 4, *body arching and lifting of pelvic structures*.

Sixteen hours before the experiment, food was removed from the cage (rats were not allowed to eat but could drink water), and the 8F catheter with the balloon (lubricated with paraffin oil) was inserted through the anus until the balloon remained in the colon 7.0 cm from the anus. The catheter was fixed at the tail root of the rat at 1.0 cm outside the anus, and rats were placed in the fixator which enabled them to only move forward and backward without the ability to turn around. After 30 min of adaptation, rats were gradually injected with normal saline at 25°C. When the AWR score of rats was 3 (the abdominal and back muscles contracted strongly, and the abdomen was lifted off the ground), the injection was stopped, and the volume of the injected liquid was read once every 3 min, repeated three times, and the average value was taken.

### 2.7. Sample Collection

After the last intervention, the rats in each group were fasted but were allowed to drink water for 12 h. Then, rats were anesthetized with urethane (1 g/kg) by intraperitoneal injection, and about 100 mg fecal samples were collected from the colon under sterile conditions, placed in an enzyme-free EP tube, and stored in a −80°C ultralow temperature refrigerator.

### 2.8. High-Throughput Sequencing and Data Analysis

The fecal samples of each group were selected for 16S rRNA gene V4-V5 region-specific primers to amplify specific regions. The primer sequences 341F (5⁣′-CCTAYGGGRBGCASCAG-3⁣′) and 806R (5⁣′-GGACTACNNGGGTATCTAAT-3⁣′) were selected for PCR amplification, and an about 420 bp amplified fragment was obtained. The 2 × 250 bp paired-end data were obtained by sequencing on the Illumina NovaSeq 6000 platform. The raw data were spliced, and the original sequencing sequence was quality-controlled by PRINSEQ software spliced by PANDAseq software. Under the 97% similarity of VSEARCH software, the chimera was removed by combining denovo and Uchime through the SILVA rRNA database (release_132), and OTU (operational taxonomic unit) clustering and species classification analysis were performed. The software QIIME was used to select a sequence with the highest abundance as a representative, and the UCLUST method was used to align the representative sequence to the SILVA rRNA database for species annotation. Alpha and beta diversity analysis, microbiota species composition, and difference analysis were performed using R software (Synbio Technologies, Suzhou, China, Project No. TN2078).

### 2.9. Statistical Analysis

SPSS 21.0 statistical software was used for analysis. The measurement data were expressed as *x* ± *s*, and a one-way variance analysis was used to compare multiple groups. *t*-test and the Wilcox rank sum test were used to analyze the differences in diversity index and functional prediction abundance between the two groups. For species composition differences, the Kruskal-Wallis rank sum test was used for comparison between multiple groups. *p* < 0.05 was considered statistically significant.

## 3. Results

### 3.1. Changes in Visceral Sensitivity of Rats in Each Group

When the rat AWR test score was 3, a lower quantity of fluid volume and higher visceral sensitivity were observed. Compared with the ZC group, the visceral sensitivity of rats in the other groups significantly increased (*p* < 0.01), except for PX1. The difference between PX3 and PX4 was significant (*p* < 0.01). Also, the visceral sensitivity of the GY2 group was significantly higher than that of the GY1 group (*p* < 0.01). Compared with the PX3 and GY1 groups, the visceral sensitivity of the GYPX1 group was significantly increased (*p* < 0.01). Compared with the PX4 and GY2 groups, the visceral sensitivity of the GYPX2 group was significantly increased (*p* < 0.05, *p* < 0.01). There was also a significant difference in visceral sensitivity between GYPX1 and GYPX2 groups (*p* < 0.05) (Table 1).

### 3.2. Gut Microbiota Species Annotation and Evaluation

**Figure 1:**
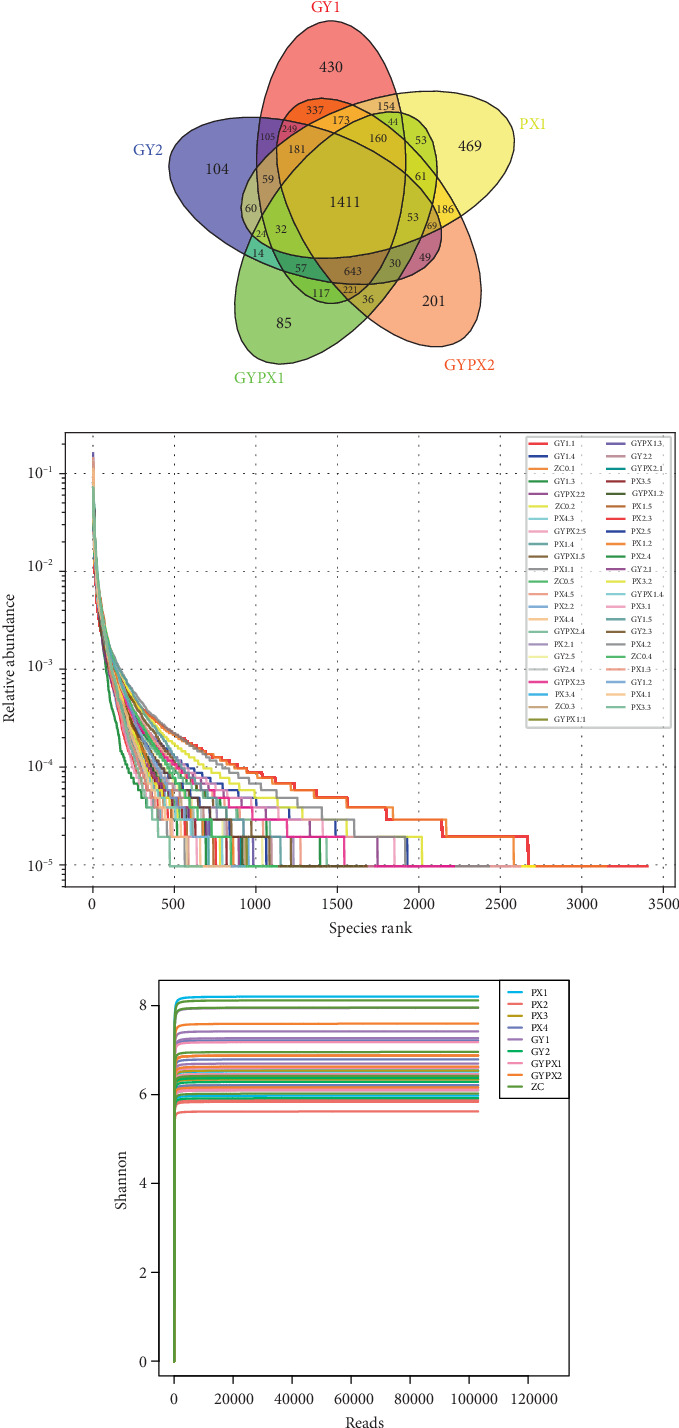
Annotation and evaluation of gut microbiota in each group.

The analysis of OTU species after leveling according to the minimum number of sample sequences showed that there were 45 samples in 9 groups of sequencing data, and the length of the microbiota sequence was 400~500 bp, which was consistent with the length of the 16S rRNA V3-V4 region sequence. According to the 97% similarity clustering classification, 6443 OTUs were obtained. There were 1411 same OTUs in five groups, 2720 OTUs (unique 469) in the PX1 group, 3300 OTUs (unique 430) in the GY1 group, 3036 OTUs (unique 104) in the GY2 group, 2956 OTUs (unique 85) in the GYPX1 group, and 3859 OTUs (unique 201) in the GYX2 group (Figure 1a).

The rank abundance curve can be used to explain the richness and evenness of species composition. The results showed that the distribution length of each group on the horizontal axis was wider, and the curve trend in the vertical axis was flat, suggesting that the richness and evenness of gut microbiota species composition were higher (Figure 1b). A certain amount of sequencing data was randomly selected from the sample, and the number of species or diversity index represented by it was counted; then, the rarefaction curve was drawn. The results showed that the Shannon dilution curve tended to be flat, indicating that the amount of sequencing data was gradually reasonable, reflecting the biodiversity information of most gut microbiota in the sample (Figure 1c).

### 3.3. Alpha Diversity Analysis

**Figure 2:**
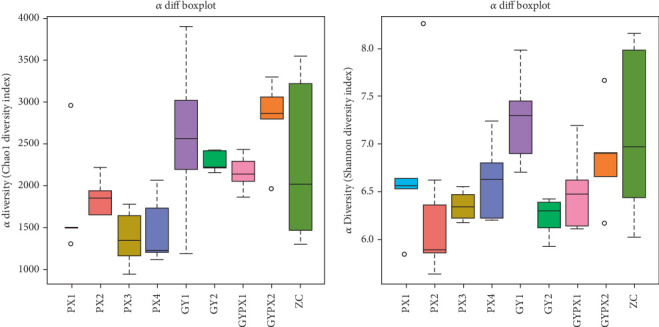
Alpha diversity of gut microbiota in each group (*x* ± *s*, *n* = 5).

Alpha diversity is the analysis of species diversity in the sample, including the richness and evenness of species composition. The Chao1 index reflects the richness of the colony in the sample, and the Shannon index reflects the community's diversity. The Chao1 index of the PX3 and PX4 groups was significantly lower than that of the ZC group (all *p* < 0.05). Also, the Chao1 index was significantly different between GYPX1 and PX3 (*p* < 0.01), between GYPX2 and PX4 (*p* < 0.01), and between GYPX1 and GYPX2 (*p* < 0.01). The Shannon index of the PX2 group was significantly lower than that of the normal group (*p* < 0.05). In addition, the Shannon index was significantly different between GY1 and GY2 (*p* < 0.05), between GY1 and GYPX1 (*p* < 0.05), and between GY2 and GYPX2 (*p* < 0.05), which suggested that senna leaf and restraint stress have different characteristics in the diversity of gut microbiota in D-IBS rats (Figure 2).

### 3.4. Beta Diversity Analysis of Gut Microbiota

**Figure 3:**
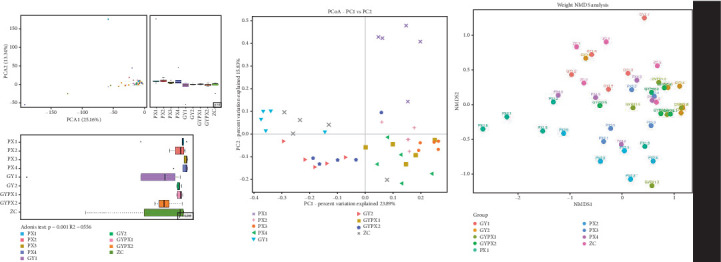
Beta diversity of gut microbiota in each group.

Beta diversity was used to compare the species diversity of gut microbiota among different samples so as to explore the difference in microbiota composition. Adonis analysis showed that the difference between groups was greater than that within groups (*R* = 0.556, *p* = 0.001), suggesting that the structure of gut microbiota in each group was different. The results of principal coordinate analysis (PCoA) and nonmetric multidimensional scaling (NMDS) showed that the PX (1–4) group, GY2 group, and GYPX (1–2) group were separated from the ZC group, suggesting that the gut microbiota structure of D-IBS rats with spleen deficiency, liver depression, and with both liver depression and spleen deficiency was altered. The gut microbiota structure in the GYPX2 group was similar to the GY2, PX4, and GYPX1 groups but different from the PX3 and GY1 groups. It is suggested that senna leaf, restraint stress, and the combination of the two factors all regulate the composition and structural changes of gut microbiota in D-IBS rats to a certain extent, and the combination of the two factors is better (Figure 3).

### 3.5. Species Composition Analysis

The species community histogram was used to compare the dominant species and their relative abundance of gut microbiota in different samples. The results showed that the gut microbiota of rats in each group had certain differences at the level of phylum, class, order, family, genus, and species. At the phylum level, the dominant species with differences in the gut microbiota of each group of rats were mainly attributed to Bacteroidetes, Firmicutes, Proteobacteria, Tenericutes, Acidobacteria, and so forth, with the highest proportion of Bacteroidetes and Firmicutes. The relative abundance ratio of Bacteroidetes was the highest in the GYPX1 group (65.31%), followed by the GY2 group (64.67%), GYPX2 group (62.37%), PX3 group (57.24%), PX2 group (53.87%), ZC group (52.66%), PX4 group (51.84%), GY1 group (49.31%), and PX1 group (37.18%). The relative abundance of Firmicutes was the highest in the PX1 group (56.45%), followed by the PX4 group (42.97%), GY1 group (41.40%), ZC group (40.57%), PX2 group (39.61%), PX3 group (36.95%), GY2 group (31.57%), GYPX2 group (30.85%), and GYPX1 group (29.20%).

**Figure 4:**
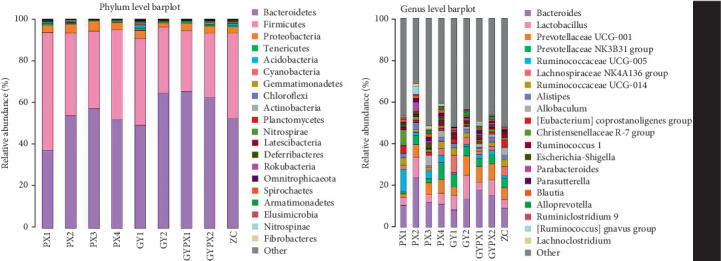
Relative abundance of gut microbiota at phylum and genus levels in each group (*x* ± *s*, *n* = 5).

Bacteroides showed a higher relative abundance at the genus level in each group. The relative abundance ratio of Bacteroides was the highest in the PX2 group (23.69%), followed by GYPX1 group (17.70%), GYPX2 group (15.11%), GY2 group (13.56%), PX3 group (11.75%), PX4 group (10.99%), PX1 group (10.55%), ZC group (9.13%), and GY1 group (8.19%) (Figure 4).

### 3.6. Linear Discriminant Analysis Effect Size (LEfSe) Multilevel Species Discriminant Analysis

**Figure 5:**
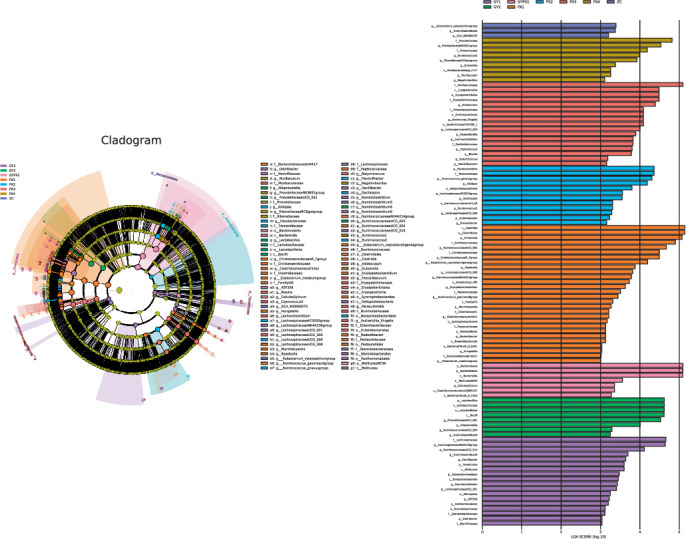
LEfSe multilevel species discriminant analysis of gut microbiota in each group.

LEfSe was used to analyze the changes in microbiota from phylum to the genus in each group of rats, and the species with significant differences were analyzed (Figure 5). The LDA threshold was set to three. The results of LEfSe analysis showed species with significant differences in abundance in the gut microbiota of rats in each group. The differential microbiota in the ZC group were *Eubacterium_xylanophilum* group, *Ruminiclostridium5*, and *GCA_900066575*. The differential microbiota in the PX1 group were Clostridia, Clostridiales, Firmicutes, Ruminococcaceae, and so forth. The differential microbiota of the PX2 group were *Parabacteroides*, Tannerellaceae, *Ruminococcus_gnavusgroup*, and so forth. The differential microbiota in the PX3 group were Muribaculaceae, Erysipelotrichia, Erysipelotrichales, and so forth. The differential microbiota in the PX4 group were Prevotellaceae, *Prevotellaceae NK3B31* group, Rikenellaceae, and so forth. The differential microbiota in the GY1 group were Lachnospiraceae, *Lachnospiraceae NK4A136* group, *Ruminococcaceae UCG_014*, and so forth. The differential microbiota in the GY2 group were *Lactobacillus*, Lactobacillaceae, Lactobacillales, and so forth. The differential microbiota in the GYPX1 group was Bacteroidales, Bacteroidetes, Bacteroidia, and so forth.

### 3.7. Predictive Analysis of Gut Microbiota Function

**Figure 6:**
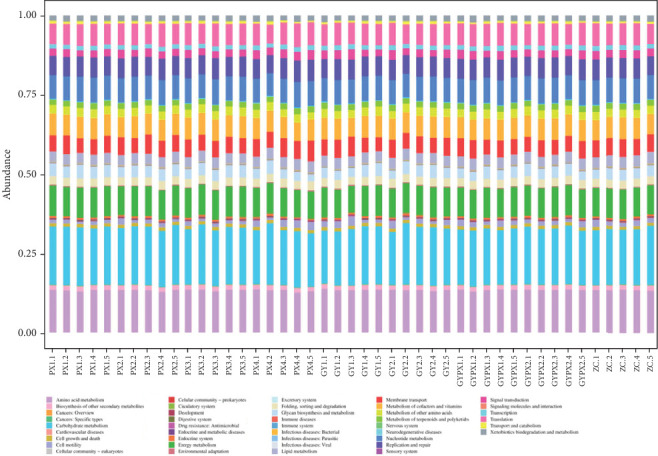
Analysis of function prediction of gut microbiota.

PICRUSt2 can be used to predict the function of gut microbiota in sample amplicon sequencing results. The KEGG statistical results showed that the Top 42 functional pathways of total abundance were taken at the secondary pathway level. The gut microbiota of each group was mainly related to amino acid metabolism, carbohydrate metabolism, specific types of cancers, endocrine system, replication and repair, nucleotide metabolism, and other pathways, as shown in Figure 6.

## 4. Discussion

Currently, the D-IBS liver depression and spleen deficiency disease combination animal model is an evidence model constructed based on the disease model of modern medical theory, further following the TCM theory, which has not yet been standardized. Common modeling methods include mother-infant separation, restraint stress, senna gavage, and acetic acid enema or the combined application of several factors [6], all of which can reproduce an animal model that meets a certain symptom or disease mechanism of D-IBS, which is similar to that of human D-IBS disease evidence. The “senna administration” and “restraint stress” are common inducing factors in the modeling of D-IBS [7–9], and the performance and detection indexes of the animal model after composite two-factor superimposed modeling coincide with the characteristics of the clinical D-IBS syndrome of liver depression and spleen deficiency to a higher degree [10]. So the rat model of D-IBS with liver depression and spleen deficiency syndrome was commonly used in study of revealing the efficacy and mechanism of TCM formulas, but there is little research on the pathological changes, the respective roles of liver depression and spleen deficiency factors, and the interaction between the two in the formation process of D-IBS liver depression and spleen deficiency syndrome. In the early stage, we also use this model to study the efficacy and mechanism of the Jianpi Huashi prescription from the perspective of the brain–gut axis and partially involved changes in the brain–gut axis function during the formation of liver depression and spleen deficiency syndrome. According to the preliminary experiments, compared with the normal control group, rats in the model group had a significantly higher grade of loose stools, a significantly higher index of colonic motility, a significantly lower sugar-water preference, a significantly higher content of colonic 5-HT and CRH, and a significant decrease in the expression of colonic tight junction proteins, AQP3, and AQP4 proteins, after given senna for 5 h [11]. It indicates that rats in the model group have abnormal colonic motor and sensory functions, elevated visceral sensitivity, depression and anxiety manifestations, diarrhea symptoms, loose and unshaped stools, impaired colonic barrier function, and imbalance of water absorption and secretion, and these indexes are basically in line with the current commonly used evaluation criteria for clinical evaluation of Chinese and Western medicine D-IBS liver depression and spleen deficiency syndrome. However, in the process of the formation of liver depression and spleen deficiency syndrome in the dual factor model, the effects of spleen deficiency and liver depression factors on the brain–gut axis function have their own characteristics, which is not completely consistent with the TCM theory that “liver depression factors aggravate spleen deficiency symptoms,” indicating the complexity of the dual factor model.

In the theory of Chinese medicine, the liver and spleen have a close relationship with each other in terms of physiological and pathological coordination and constraints. As mentioned in the Yellow Emperor's Inner Canon, “untreated, the liver is transmitted to the spleen,” which puts forward the important proposition that liver disease is transmitted to the spleen. Zhang Jingyue of the Ming Dynasty clearly expressed the etiology of liver depression and spleen deficiency as follows: “If anger harms the liver, the qi of the liver will invade the spleen, and if the qi of the stomach is injured, it will prevent diet. Although this is caused by the reversal of liver qi, the liver qi will gradually dissipate, and the damage to the temperament will be affected by it. Therefore, it is not necessary to focus on the liver, but on the spleen.” He also said, “the pathogenic factor of the five organs is all connected to the spleen and stomach. If the pathogenic factor of the liver invades the spleen, both the liver and spleen are all empirical, and the liver is strong and the spleen is weak. Sacrificing the liver and saving the spleen is also possible.” It was pointed out that the main cause of liver depression and spleen deficiency syndrome is the damage to the liver caused by depression and anger, the loss of liver circulation, and the transverse reversal of the spleen [12].

Based on the combination of the above two factors, domestic scholars established a D-IBS rat model of liver depression and spleen deficiency syndrome by combining the method of restraint stress (2 weeks) based on intragastric administration of senna leaf for 2 weeks. Therefore, in this experiment, the rat model of D-IBS with liver depression and spleen deficiency syndrome was established by intragastric administration of senna leaf combined with restraint stress stimulation. At the same time, the rat model of D-IBS with spleen deficiency syndrome was established by intragastric administration of senna leaf only, and the rat model of D-IBS with liver depression syndrome was established by restraint stress stimulation only. It was preliminarily confirmed that with the prolongation of senna leaf administration, the visceral sensitivity of D-IBS rats with spleen deficiency syndrome showed an increasing trend, especially significant differences between the PX3 and PX4 groups. The liver depression group also showed the same trend. The visceral sensitivity of the D-IBS model rats with liver depression and spleen deficiency syndrome established by the combined application of the two factors was significantly higher than that of the one-factor model at the same time, and the difference was significant. The results are consistent with the theory of TCM, which implies that “liver depression can aggravate spleen deficiency symptoms.”

Increased visceral sensitivity is one of the important pathological mechanisms of D-IBS [13]. Studies have shown that under low levels of inflammation or chronic stress stimulation, the intestinal tract can cause hypothalamus–pituitary–adrenal axis hyperfunction, thereby increasing visceral sensitivity [14]. The senna leaves are cold-natured and belong to the purgative medicine. The sennoside contained in it can significantly reduce the absorption of water and electrolytes in the intestine and significantly increase the secretion of body fluids into the intestine. It can promote the release of motilin in the intestine, reduce the somatostatin level in the intestine, and inhibit the activity of Na + -K + -ATPase in the intestinal mucosa, resulting in abdominal pain and diarrhea [15]. Because of its cold nature, long-term use can hurt the spleen. Williams et al. [16] used the restraint stress method to establish the D-IBS rat model. Through the restraint stimulation, the emotional changes of the rats were caused, which affected the gastrointestinal function and caused abdominal pain and soft stool. The theoretical basis of this method is consistent with the theory of liver depression in TCM. According to the TCM theory, the liver belongs to wood, and the spleen belongs to soil. In the case of spleen deficiency, emotional stimulation or liver depression can aggravate the symptoms of spleen deficiency.

In recent years, various studies have found that gut microbes can interact with the brain through the brain–gut axis. The Yellow Emperor's Internal Classic (Suwen) suggests that the stagnation of liver qi needs to be relieved to restore qi, which is consistent with the mechanism of regulating the HPA axis and neurotransmitters in modern research to improve the imbalance of bacteria. For example, IBS patients may experience excessive proliferation of pathogenic bacteria, a decrease in the proportion of probiotics such as lactic acid bacteria and bifidobacteria, disruption of gut microbiota leading to hyperfunction of the HPA axis, and disorder in the regulation of brain–gut peptides such as 5-HT, CRH, and substance P (SP). The gut microbiota of patients with depression is different from that of the normal population [17]. According to Jingyue Quanshu, the spleen and stomach are the foundation of postnatal development, the main transportation of water and grains, and the source of qi and blood biochemistry. Spleen deficiency leads to abnormal circulation, resulting in insufficient qi and blood and affecting the balance of intestinal microbiota. Research has shown that spleen deficiency can affect the species richness and quantity of gut microbiota, and the structure and diversity of gut microbiota in rats with spleen deficiency diarrhea significantly decrease. The gut microbiota affects the function of the spleen by regulating the immune system, neuroendocrine system, and so forth. Spleen deficiency can also affect the structure and function of the gut microbiota through the brain–gut axis [18]. Dysfunction of the spleen and soil transport is caused by the spleen and stomach. Patients often experience recurrent intestinal symptoms, leading to anxiety, depression, and other emotions, which in turn cause liver dysfunction and further exacerbate intestinal symptoms. Psychological factors (liver wood) and intestinal function (spleen soil) mutually influence and exacerbate each other, thus forming a vicious cycle.

Gut microbiota has an important role in human health and disease. With the launch of the Human Microbiome Project (HMP) and the Metagenomics of the Human Intestinal Tract (MetaHIT) project, the regulation of gut microbiota on gastrointestinal function and maintaining gastrointestinal homeostasis has been extensively investigated. Current research focuses on the effects of small intestinal bacterial overgrowth (SIBO) and changes in gut microbiota diversity on IBS [19]. The study found that the frequency of defecation may be related to mucosal *Lactobacillus* and *Bifidobacterium*. *Escherichia coli* and *Enterococcus* are also invasive under certain conditions [20]. The diversity of gut microbiota was significantly lower than normal [21, 22].

There is an important interaction between gut microbiota and the brain–gut axis, which regulates gastrointestinal nerve, endocrine, and immune function and maintains the homeostasis of the digestive tract [23]. Studies have shown that gut microbiota as the main target can change gastrointestinal motility and reduce visceral sensitivity, thereby alleviating the clinical symptoms of D-IBS [24, 25]. This study used 16S rRNA high-throughput sequencing technology to detect gut microbiota diversity in D-IBS model rats with liver depression and/or spleen deficiency syndrome. It was found that senna leaf, restraint stress, and the combination of the two factors could affect the gut microbiota of rats, change the diversity of microbiota, and affect the structure and composition of microbiota. Therefore, gut microbiota may be one of the important mechanisms for the occurrence of different syndromes of D-IBS.

The gut microbiota diversity analysis results showed that compared with normal rats, the Chao1 index of rats in the PX group showed a downward trend, and the difference between the PX3 and PX4 groups was significant. The GY and GYPX groups showed an upward trend, but no significant difference existed. The GYPX1 and GYPX2 groups were significantly higher than the PX3 and PX4 groups, respectively. Except for the GY1 group, the Shannon index of the other groups decreased to varying degrees compared with the normal group, and the difference between the PX2 group was significant. There was a significant difference in the Shannon index between the GY1 and GY2 groups. The GYPX1 and GYPX2 groups were also significantly different from GY1 and GY2, respectively. It is indicated that senna, restraint stress, and the combination of the two factors have a certain degree of influence on the structure and quantity of gut microbiota in D-IBS rats, destroying the dynamic balance of the microbiota, indicating that senna and restraint stress have different characteristics in changing the abundance and diversity of the microbiota, and the changes in the abundance and diversity of the microbiota after the combined application of the two factors are also inconsistent. Considering that there are only five samples in each group in the experiment, it is necessary to further verify. PCoA analysis and NMDS analysis showed that the aggregation groups of the PX (1–4) group and GY2 group and GYPX (1–2) group were far away from the ZC group, while those of the GYPX2 group were closer to the GY2 group and PX4 group and closer to the GYPX1 group but far away from the PX3 and GY1 group, indicating that senna leaf, restraint stress, and the combination of the two factors all affected the composition and structure of gut microbiota in D-IBS rats to a certain extent, and the combination of the two factors had a greater impact.

The species composition of gut microbiota and LEfSe multilevel species difference analysis showed that at the phylum level, Bacteroidota and Firmicutes accounted for the highest proportion of the gut microbiota of rats in each group. Studies have shown that Bacteroidetes can bind to lipopolysaccharide-binding proteins and cluster of differentiation 14 (CD14) molecules on the cell surface, thereby activating the nuclear factor kappa B (NF-*κ*B) signaling pathway and promoting the expression of various proinflammatory factors. Decreased abundance of Bacteroidetes has been observed in spleen yang vacuity model rats [26]. On the other hand, Firmicutes can maintain the balance in gut microbiota by accelerating glycolysis [27]. The increased abundance of Bacteroidetes and the decreased abundance of Firmicutes are closely related to the occurrence of intestinal inflammation [28, 29]. This experiment showed the highest proportion of Bacteroidetes in the GYPX1 group, and the lowest proportion of Firmicutes was in the GYPX1 group, indicating that senna combined with restraint stress could cause gut microbiota disorder. At the genus level, Bacteroides had the highest relative abundance in each group. Bacteroides is a nonspore anaerobic bacterium that can promote protein and fat decomposition, and its abundance is negatively correlated with intestinal inflammation [30]. The results showed the highest relative abundance in the GYPX1 group, indicating that the D-IBS liver stagnation and spleen deficiency syndrome model established by gavage of senna combined with restraint stress was a noninflammatory model.

The results of PICRUSt2 function prediction analysis showed that the gut microbiota of each group was mainly related to various metabolic pathways such as amino acid metabolism and carbohydrate metabolism. Amino acids and their metabolites have an important biological role in gastrointestinal diseases, such as enhancing the expression of tight junction proteins and intestinal mucosal barrier function, reducing intestinal inflammation by regulating the ubiquitin protease system [31], and directly improving the intestinal microecological environment [32]. Carbohydrates are the main carbon source, whose effective intake can increase human beneficial bacteria and reduce pathogenic bacteria, thereby restoring the balance of gut microbiota and promoting the production of various short-chain fatty acids (SCFAs) [33, 34]. SCFAs can protect the intestinal mucosa, regulate the body's immunity, and reduce the intestinal inflammatory response [35, 36].

The functional prediction results show that the combination of senna and restraint stress may regulate the gut microbiota of D-IBS by improving the amino acid metabolism, carbohydrate metabolism, and other metabolic pathways of the gut microbiota. However, how to improve the gut microbiota and make the rats become D-IBS liver depression and spleen deficiency syndrome through the above metabolic pathways by intragastric administration of senna combined with restraint stress should be further studied and explored based on the molecular biology level and cell level.

Liver depression and spleen deficiency syndrome refers to liver qi stagnation, dysfunction of detachment, and excretion, resulting in the spleen's loss of health and transportation, which leads to a series of digestive symptoms, such as abdominal distension, belching, loss of appetite, and diarrhea, which are closely related to both the digestive system and psychopsychological factors. Therefore, the present model induced liver depression syndrome by binding stress, and senna caused diarrhea to simulate spleen deficiency and diarrhea, which did not exclude the complications of D-IBS and the future need to design a hierarchical modeling to differentiate between single syndrome and comorbidities. A study on the characteristics of intestinal flora in different Chinese medicine certificates of diarrhea-type irritable bowel syndrome by some scholars found that the highest diversity of flora was found in the healthy control group, followed by the group of liver depression and spleen deficiency, and the lowest in the group of spleen-kidney yang-deficiency. There were significant differences in the composition of the flora among the three groups, with Bifidobacteriaceae and Ruminococcaceae enriched in the control group; Enterobacteriaceae, Clostridiaceae, and Aminoacidococcaceae in higher abundance in the spleen-kidney yang-deficiency group; and *Streptococcus* as a characteristic bacterium in the liver-depression-spleen-deficiency group. The study confirmed that there was a correlation between TCM patterns and intestinal flora, and different patterns had specific flora characteristics [37]. In this study, the diversity of bacterial flora was different in the spleen deficiency group, the liver depression group, and the liver depression and spleen deficiency group, indicating that different TCM evidence types have different effects on the intestinal flora, with the Bacteroidota elevated in the modeling group, the Firmicutes decreased in the modeling group, and the abundance of pathogenic bacteria, such as Bacteroidales, increased in the spleen deficiency and liver depression group. This study revealed the correlation between gut microbiota and D-IBS and provided a basis for the targeted regulation of intestinal flora for the symptomatic treatment of D-IBS with liver depression and spleen deficiency, such as inhibiting Bacteroidota and supplementing thick-walled Bacteroidales. But in this experiment, only 16S rRNA sequencing was used for the study of intestinal flora, which has some limitations, such as only analyzing the structure of the flora, unable to analyze the functional genes and metabolic pathways, and the selection of primers may have missed the detection of some flora; the causal relationship between gut microbiota and D-IBS could not be further verified through experiments, and it was not clear whether the change of intestinal microbiota was the cause or the consequence of D-IBS. In the future, follow-up studies will combine macrogenome sequencing and metabolomics to reveal the functional pathways of intestinal flora or use fecal microbiota transplantation and other interventions to further explore the causal relationship between intestinal microbiota and D-IBS, so as to provide a more solid experimental basis for elucidating the pathogenesis of D-IBS and D-IBS. It confirms that there is an objective correlation between the TCM evidence and the composition of intestinal flora, which provides a scientific basis for “understanding TCM evidence from the perspective of flora.” In the future, probiotic therapy and fecal flora transplantation can be used to specifically inhibit or supplement a certain flora to achieve the purpose of treating different evidence types of D-IBS.

## 5. Conclusion

In summary, senna combined with restraint stress has different effects on visceral hypersensitivity, microbiota composition, and metabolic pathways in D-IBS rats with liver depression and spleen deficiency syndrome. The experimental results suggest that the changes in gut microbiota structure and its metabolic pathway may be an important biological basis of D-IBS liver depression and spleen deficiency syndrome, which provides a new idea for clinical prevention and treatment. In addition, these data suggest that the TCM pattern of liver depression and spleen deficiency is correlated with the gut microbiota and that the factor of liver depression plays an important role, which may aggravate the symptoms of spleen deficiency and thus lead to the pathogenesis of liver depression and spleen deficiency, thus further refining the TCM theory and the pathogenesis of liver depression and spleen deficiency in D-IBS. However, due to the limitations of experimental conditions and technical resources, this study has some limitations and failed to fully detect and explain the composition and mechanism of gut microbiota involved in the pathogenesis of D-IBS. Therefore, further studies are needed to investigate the specific mechanisms of gut microbiota involved in D-IBS.

## Figures and Tables

**Table 1:** Changes in visceral sensitivity of rats in each group (*x* ± *s*, *n* = 10).

**Group**	**The water volume (mL) when the AWR test score was 3**
ZC	0.81 ± 0.08
PX1	0.75 ± 0.13
PX2	0.66 ± 0.07^∗^
PX3	0.57 ± 0.03^∗^
PX4	0.51 ± 0.02^∗^^·^
GY1	0.79 ± 0.04^∗^
GY2	0.59 ± 0.09^∗^^*Δ*^
GYPX1	0.50 ± 0.04^∗^^*Δ*·^
GYPX2	0.46 ± 0.07^∗^^#※⁜^

⁣^∗^*p* < 0.01 versus ZC group; ^*Δ*^*p* < 0.01 versus GY1 group; ^#^*p* < 0.01 versus GY2 group; ^·^*p* < 0.01 versus PX3 group; ^※^*p* < 0.05 versus PX4 group; ^⁜^*p* < 0.05 versus GYPX1 group.

## Data Availability

All data generated or analyzed during this study are included in this published article.

## References

[1] Simrén M., Törnblom H., Palsson O. S., Whitehead W. E. (2017). Management of the Multiple Symptoms of Irritable Bowel Syndrome. *The Lancet Gastroenterology & Hepatology*.

[2] Slim M., Calandre E. P., Rico-Villademoros F. (2015). An Insight Into the Gastrointestinal Component of Fibromyalgia: Clinical Manifestations and Potential Underlying Mechanisms. *Rheumatology International*.

[3] Rhee S. H., Pothoulakis C., Mayer E. A. (2009). Principles and Clinical Implications of the Brain-Gut-Enteric Microbiota Axis. *Nature Reviews Gastroenterology & Hepatology*.

[4] Bonaz B., Bazin T., Pellissier S. (2018). The Vagus Nerve at the Interface of the Microbiota-Gut-Brain Axis. *Frontiers in Neuroscience*.

[5] Wang H., Zeng A., Cao R., Guo Z. H., He Y. S., Tan Z. J. (2014). Mechanisms Underlying Regulatory Effects of Qiweibaizhusan on Intestinal Microecology. *World Chinese Journal of Digestology*.

[6] Wu X. L., Sun J. H. (2010). Irritable Bowel Syndrome: Current State of Experimental Research in China. *World Chinese Journal of Digestology*.

[7] Zhai J. H., Chen L., Xu S. (2022). Mechanism Study of Yuan’s Spleen-Supporting and Cleansing Formula for the Treatment of Diarrhea-Type Irritable Bowel Syndrome by Regulating miR-199a/TLR4/NF-*κ*B Signaling Pathway. *Journal of Chinese Medicinal Materials*.

[8] Liu T., Wu M. L., Deng G. Y. (2023). Linderae Radix Water Extract Treats Diarrhea-Predominant Irritable Bowel Syndrome in Rats: A Serum Metabolomics Study. *China Journal of Chinese Materia Medica*.

[9] Qiu S. K., Kong L. D., Yuan J. Y., Zheng Y. (2018). Effects of Changqi Drink on Brain-Gut Peptides in Diarrhea-Predominant Irritable Bowel Syndrome Rats With Syndrome of Liver Stagnation and Spleen Deficiency. *Shanghai Journal of Traditional Chinese Medicine*.

[10] Jiang F. R., He Y. C., Wu Y. (2024). Fitting Degrees of Animal Models of Diarrhea-Irritable Bowel Syndrome With Clinical Characteristics of Western Medicine and Traditional Chinese Medicine. *Chinese Journal of Experimental Traditional Medical Formulae*.

[11] Liu Z. W., Yu J. J., Ma X. R. (2023). Changes of Colonic Tight Junction Proteins, AQP3 and AQO4 in Rats With Diarrhea-Type IBS Model of Liver-Depression and Spleen-Deficiency Syndrome. *Chinese Journal of Gerontology*.

[12] Liu Y., Ding X. F., Yan Z. W. (2018). Discussion on the Basic Theory of Traditional Chinese Medicine of Syndrome of Stagnation of Liver Qi and Spleen Deficiency. *China Journal of Traditional Chinese Medicine and Pharmacy*.

[13] Tang Y., Chen A., Chen Y., Guo L., Lin C. (2016). Zeta Inhibitory Peptide as a Novel Therapy to Control Chronic Visceral Hypersensitivity in a Rat Model. *PLoS One*.

[14] Salyers M. P., Bonfils K. A., Luther L. (2017). The Relationship Between Professional Burnout and Quality and Safety in Healthcare: A Meta-Analysis. *Journal of General Internal Medicine*.

[15] Sun S. F., Zhang Y. Y. (2017). Research Progress on Components and Pharmacological Effects of Senna Leaves. *Shandong Chemical Industry*.

[16] Williams C. L., Villar R. G., Peterson J. M., Burks T. F. (1988). Stress-Induced Changes in Intestinal Transit in the Rat: A Model for Irritable Bowel Syndrome. *Gastroenterology*.

[17] Wang H., Wang S. X. (2020). Discussion of Correlation Between Chronic Psychological Stress Induced Liver Depression and Spleen Deficiency Syndrome and Microbiota-Gut-Brain Axis and Study on Intervention Mechanism of Xiaoyaosan. *Chinese Journal of Experimental Traditional Medical Formulae*.

[18] Yun X. W., Wu F. Z., Xu Y. F. (2023). Exploration of Insomnia of the Liver Constraint and Spleen Deficiency Pattern From the Perspective of Gut Microbiota-Intestine-Brain Axis. *Modern Chinese Clinical Medicine*.

[19] Yin X. L., Wang F. Y., Tian Y. X., Duan Y. Z., Tang X. D. (2017). Correlation Between Intestinal Flora Imbalance and Brain-Gut Axis Dysregulation in Irritable Bowel Syndrome. *Proceedings of the 29th National Conference on Integrated Traditional Chinese and Western Medicine Digestive System Diseases*.

[20] Parkes G. C., Rayment N. B., Hudspith B. N. (2012). Distinct Microbial Populations Exist in the Mucosa-Associated Microbiota of Sub-Groups of Irritable Bowel Syndrome. *Neurogastroenterology and Motility*.

[21] Guo H. L., Shao Y. Y., Meng H., Bi L. G., Zhang H. P. (2015). Research on the Relation Between Gastrointestinal Microbiota and Disease. *Microbiology China*.

[22] Meng L. Y., Chen X. Q., Shi D. Y., Huang H. D., Ggo S. N. (2013). Influence of Sijunzi Decoction on Intestinal Flora Diversity in Spleen-Deficient Rats. *Chinese Journal of Animal & Veterinary Ences*.

[23] Yan S. G., Wang L. S. (2017). Stress and Gut Microbiota Interaction in the Regulation of Visceral Pain of IBS. *Chinese Journal of Microecology*.

[24] Distrutti E., Monaldi L., Ricci P., Fiorucci S. (2016). Gut Microbiota Role in Irritable Bowel Syndrome: New Therapeutic Strategies. *World Journal of Gastroenterology*.

[25] El-Salhy M., Hatlebakk J. G., Hausken T. (2019). Diet in Irritable Bowel Syndrome (IBS): Interaction With Gut Microbiota and Gut Hormones. *Nutrients*.

[26] Lin X., Huang Y., Yang S. S. (2021). Effect of Fuzi Lizhong Pill on Intestinal Flora of Spleen Yang Deficiency IBS-D Rats Based on High-Throughput Sequencing Technique. *Journal of Nanjing University of Traditional Chinese Medicine*.

[27] Porter N. T., Martens E. C. (2017). The Critical Roles of Polysaccharides in Gut Microbial Ecology and Physiology. *Annual Review of Microbiology*.

[28] Burrello C., Garavaglia F., Cribiù F. M. (2018). Therapeutic Faecal Microbiota Transplantation Controls Intestinal Inflammation Through IL10 Secretion by Immune Cells. *Nature Communications*.

[29] Zhang X., Zhang D., Jia H. (2015). The Oral and Gut Microbiomes Are Perturbed in Rheumatoid Arthritis and Partly Normalized After Treatment. *Nature Medicine*.

[30] Wen B. S., Liu Y. (2021). Correlation Between Intestinal Mucosa-Associated Flora and Disease Activity in Elderly Patients With Ulcerative Colitis. *Chinese Journal of Health Medicine*.

[31] Bertrand J., Marion-Letellier R., Azhar S. (2015). Glutamine Enema Regulates Colonic Ubiquitinated Proteins but Not Proteasome Activities During TNBS-Induced Colitis Leading to Increased Mitochondrial Activity. *Proteomics*.

[32] de Souza A. Z. Z., Zambom A. Z., Abboud K. Y. (2015). Oral Supplementation With L-Glutamine Alters Gut Microbiota of Obese and Overweight Adults: A Pilot Study. *Nutrition*.

[33] AlEssa H. B., Cohen R., Malik V. S. (2018). Carbohydrate Quality and Quantity and Risk of Coronary Heart Disease Among US Women and Men. *The American Journal of Clinical Nutrition*.

[34] Tang T., Chen H. G., Zhao C., Gong X. J., Deng Q. F., Zhou X. (2021). Research Progress on Intestinal Microecology Regulating Mechanism and Biological Activities of Polysaccharides. *Zhongguo Zhong yao za zhi= Zhongguo zhongyao zazhi= China journal of Chinese materia medica*.

[35] Venegas D. P., De la Fuente M. K., Landskron G., González M. J., Hermoso M. A. (2019). Short Chain Fatty Acids (SCFAs)-Mediated Gut Epithelial and Immune Regulation and Its Relevance for Inflammatory Bowel Diseases. *Frontiers in Immunology*.

[36] Chang P. V., Hao L., Offermanns S., Medzhitov R. (2014). The Microbial Metabolite Butyrate Regulates Intestinal Macrophage Function via Histone Deacetylase Inhibition. *Proceedings of the National Academy of Sciences of the United States of America*.

[37] Chao G., Zhang S. (2020). The Characteristics of Intestinal Flora of IBS-D With Different Syndromes. *Immunity, Inflammation and Disease*.

